# A Comparative Analysis of Radical Scavenging, Antifungal and Enzyme Inhibition Activity of 3′-8″-Biflavones and Their Monomeric Subunits

**DOI:** 10.3390/antiox12101854

**Published:** 2023-10-12

**Authors:** Iva Jurčević Šangut, Bojan Šarkanj, Erna Karalija, Dunja Šamec

**Affiliations:** 1 Department of Food Technology, University North, 48000 Koprivnica, Croatia; ijurcevic@unin.hr (I.J.Š.); bsarkanj@unin.hr (B.Š.); 2 Department for Biology, Faculty of Science, University of Sarajevo, 71000 Sarajevo, Bosnia and Herzegovina; erna.k@pmf.unsa.ba

**Keywords:** 3′-8″-biflavones, amentoflavone, bilobetin, ginkgetin, isoginkgetin, sciadopitysin, apigenin, genkwanin, acacetin

## Abstract

Biflavonoids are dimeric forms of flavonoids that have recently gained importance as an effective new scaffold for drug discovery. In particular, 3′-8″-biflavones exhibit antiviral and antimicrobial activity and are promising molecules for the treatment of neurodegenerative and metabolic diseases as well as cancer therapies. In the present study, we directly compared 3′-8″-biflavones (amentoflavone, bilobetin, ginkgetin, isoginkgetin, and sciadopitysin) and their monomeric subunits (apigenin, genkwanin, and acacetin) and evaluated their radical scavenging activity (with DPPH), antifungal activity against mycotoxigenic fungi (*Alternaria alternata*, *Aspergillus flavus*, *Aspergillus ochraceus*, *Fusarium graminearum*, and *Fusarium verticillioides*), and inhibitory activity on enzymes (acetylcholinesterase, tyrosinase, α-amylase, and α-glucosidase). All the tested compounds showed weak radical scavenging activity, while antifungal activity strongly depended on the tested concentration and fungal species. Biflavonoids, especially ginkgetin and isoginkgetin, proved to be potent acetylcholinesterase inhibitors, whereas monomeric flavonoids showed higher tyrosinase inhibitory activity than the tested 3′-8″-biflavones. Amentoflavone proved to be a potent α-amylase and α-glucosidase inhibitor, and in general, 3′-8″-biflavones showed a stronger inhibitory potential on these enzymes than their monomeric subunits. Thus, we can conclude that 3′-8″-dimerization enhanced acetylcholinesterase, α-amylase, and α-glucosidase activities, but the activity also depends on the number of hydroxyl and methoxy groups in the structure of the compound.

## 1. Introduction

**Figure 1:**
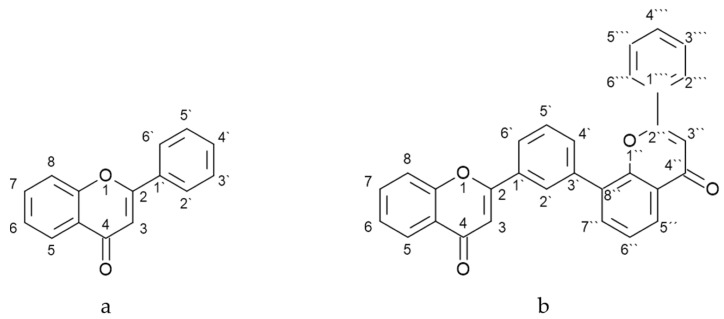
The basic structure of (**a**) flavonoids and (**b**) 3′-8″-biflavones.

Flavonoids have been the focus of scientific attention for more than 40 years and 8000 different structures have been described to date [1]. However, they are a large group of specialized metabolites whose biological function in plants and biological activity are strongly influenced by molecular structure [2]. The basic structure of flavonoids is presented in Figure 1a. In general, flavonoids occur as aglycons or in conjugated form. Aglycons are often hydroxylated at the C3, C5, C7, C3′, C4′, and C5′ positions, while some of these hydroxyl groups may also be methylated, acetylated, or sulfated [3]. Prenylation occurs directly on a carbon atom in the aromatic rings, but *O*-prenylation has also been found [3]. The hydroxylation of C5, C7, C3′, and C4′ and geranylation or prenylation at C6 have been extensively studied to increase the bacterial inhibition of flavonoids, while methoxylation at C3′ and C5 decreases the antibacterial activity of flavonoids [4]. According to a study by Zhang et al. [5], the 2,3-double bond, 4-keto groups, 3′,4′-catechol structure, and 3-hydroxyl in the flavonoid scaffold play an important role in the antioxidant behavior, while the cell proliferation assay showed low cytotoxicity for 3-*O*-methylquercetin. According to Boozari et al. [6], the prenyl group in the C8 position plays an important role in biological effects such as antimicrobial, cytotoxic, enzyme inhibitory, and estrogenic activity. The resveratrol residue (A or B ring) in combination with lavandulyl flavanones in the structure of flavonoids may enhance their cytotoxic effect, while the presence of prenyl groups in any position of the flavonoid backbone may enhance its anti-inflammatory effect [6]. The same group of authors reported that prenylated compounds with flavanone structure and hydroxyl substitution in C3 decreased antibacterial activity but had no effect on cytotoxic activity, while C8 prenylation can increase potent enzyme inhibitory activity, and this effect and C5′-prenylated chalcones with C6′-OH substitution have significant cytotoxic activity. Flavonoids can occur in free form, but in plants, they more commonly occur in conjugated form as flavonoid glycosides, which are conjugated by a bond between flavonoid aglycones and glycosyl groups. The glycosidic bond is located at position C3 or C7, and the carbohydrates are usually *L*-rhamnose, *D*-glucose, glucose–rhamnose, galactose, or arabinose [3]. The glycosylation of flavonoids can alter the biological activity of flavonoids, increase water solubility, reduce toxic effects and side effects, and improve specific targeting [7].

**Figure 2:**
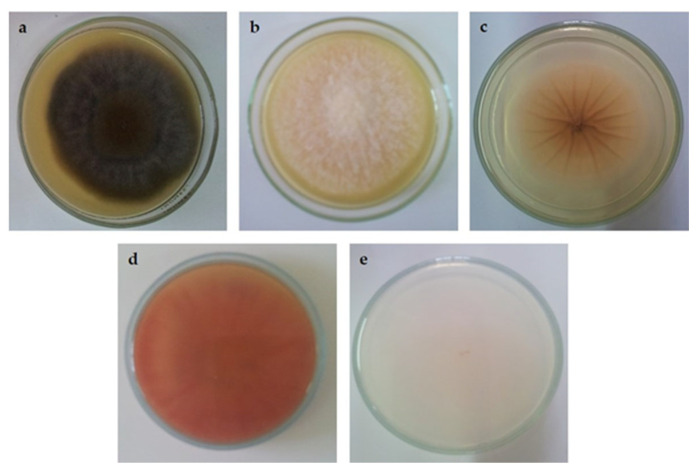
Photographs of the tested fungi without treatments: (**a**) *A. alternata*; (**b**) *A. flavus*; (**c**) *A. ochraceus*; (**d**) *F. graminearum*; (**e**) *F. verticillioides*.

Flavonoids can also occur in a dimeric form consisting of two monoflavonoid residues. The basic structure of the 3′-8″ dimer is presented in Figure 1b. To date, nearly 600 different biflavonoids have been described [8], but little is known about their biosynthesis and precise roles in plants. On the other hand, there is increasing evidence that flavonoid dimers are an effective new scaffold for drug discovery [8,9,10,11,12,13]. They have shown great potential as antimicrobial agents against viruses [14,15] and fungi [16], as well as in the treatment of neurodegenerative diseases [17] and cancers [18]. The precise mechanisms through which dimerization affects biological activity and whether this effect exists are not well understood. In this work, we aimed to investigate how the 3′-8″-dimerization affects the biological activity of flavonoids. To this end, we studied their scavenging activity (with DPPH), antifungal activity against mycotoxigenic fungi (*A. alternata*, *A. flavus*, *A. ochraceus*, *F. graminearum*, and *F. verticillioides*) (Figure 2), and inhibitory activity on enzymes (acetylcholinesterase, tyrosinase, α-amylase, and α-glucosidase) of five 3′-8″-biflavones (amentoflavone, bilobetin, ginkgetin, and isoginkgetin) and their monomeric subunits (apigenin, genkwanin, and acacetin).

## 2. Materials and Methods

### 2.1. Reagents and Standards

Amentoflavone (1) (CAS 1617-53-4), 4-nitrophenyl α-D-glucopyranoside (CAS 3767-28-0), Tris base (CAS 77-86-1), acetylcholinesterase from *Electrophorus electricus* (CAS 9000-81-1), 5,5′-dithiobis(2-nitrobenzoic acid) (CAS 69-78-3), 3,4-dihydroxy-L-phenylalanine (CAS 59-92-7), α-amylase from porcine pancreas (CAS 9000-90-2), 2,2-diphenyl-1-picrylhydrazyl (CAS 1896-66-4), 3,5-dinitrosalicylic acid (CAS 609-99-4), potassium sodium tartrate tetrahydrate (CAS 6381-59-5), starch (CAS 9005-84-9), tyrosinase from mushroom (CAS 9002-10-2), α-glucosidase from *Saccharomyces cerevisiae* (CAS 9001-42-7), 4-nitrophenyl α-D-glucopyranoside (CAS 3767-28-0), and RPMI-1640 medium were purchased from Sigma-Aldrich (St. Louis, MO, USA). Bilobetin (2) (CAS 521-32-4), isoginkgetin (3) (CAS 548-19-6), ginkgetin (4) (CAS 481-46-9), sciadopitysin (5) (CAS 521-34-6), genkwanin (7) (CAS 437-64-9), and acacetin (8) (CAS 480-44-4) were obtained from PhytoLab (Vestenbergsgreuth, Germany). Sodium phosphate, monobasic monohydrate (CAS 10049-21-5), Sodium phosphate, dibasic heptahydrate (CAS 7782-85-6), and dimethyl sulfoxide (CAS 67-68-5) were purchased from Thermo Fisher Scientific (Waltham, MA, USA). Apigenin (6) (CAS 520-36-5) was purchased from Alfa Aesar (Ward Hill, MA, USA) and S-acetylthiocholine iodide (CAS 1866-15-5) was purchased from Biosynth (Bratislava, Slovakia). Methanol (CAS 67-56-1) was obtained from Kemika (Zagreb, Croatia) and sodium hydroxide (CAS 1310-73-2) was obtained from T.T.T. (Sveta Nedjelja, Croatia).

### 2.2. DPPH Scavenging Activity

DPPH scavenging activity was determined according to the method of Brand-Williams et al. [19]. In the DPPH assay, 20 µL of each standard (1 mg/mL) was mixed with 980 µL of 0.094 mM methanolic DPPH solution. After 1 h, absorbance at 515 nm was measured, and the results are presented as a percentage of DPPH radical inhibition.

### 2.3. Antifungal Activity

The antifungal activity of the tested standards was investigated according to the method described in [20]. Fungi that were used in this experiment are major producers of mycotoxins and food contaminants—*Alternaria alternata* (WT), *Aspergillus flavus* (NRRL 3251), *Aspergillus ochraceus* (CBS 589.68), *Fusarium graminearum* (CBS 110.250), and *Fusarium verticillioides* (CBS 119.825) (Figure 2). The compounds were tested at concentrations of 0.01, 0.1, 1, and 10 μg/mL, and the results are expressed as a percentage of fungal growth.

### 2.4. Enzyme Inhibition Activity

The acetylcholinesterase inhibition assay was performed using the reaction-based assay of Ellman [21] with modifications [22]. An enzyme solution (25 µL, 0.25 U/mL), Ellman’s reagent (125 µL, 3 mM), and 50 µL of the tested compounds (100 µM) were mixed and preincubated for 15 min at room temperature. Then, 25 µL of S-acetylthiocholine iodide (15 mM) was added and incubated for 15 min at room temperature. The absorbance was measured at 405 nm using a plate reader. The blank sample was prepared in the same way with Tris-HCl buffer (50 mM, pH 8, 25 °C). The results are expressed as a percentage of inhibition.

The tyrosinase inhibition assay was performed according to Jakimiuk et al. [22], with slight modifications. The compounds (80 µL, 100 μM) were preincubated with 40 µL of a tyrosinase solution (250 U/mL) at room temperature for 10 min. Then, 80 µL of 3,4-dihydroxy-L-phenylalanine (3 mM) was added and incubated at room temperature for 10 min. The absorbance was measured at 492 nm in the plate reader. The blank was prepared using a PBS buffer (100 mM, pH 6.8, 25 °C). The results are expressed as a percentage of enzyme inhibition.

The α-amylase inhibition assay was performed according to Etsassala et al. [23], with slight modifications. In a microplate, 20 µL of each flavonoid (100 μM), 50 µL of the PBS buffer (100 mM, pH 6.8, 25 °C), and 10 µL of alpha-amylase (2 U/mL) were mixed and incubated at 37 °C for 20 min. Then, 10 µL of a 1% starch solution was added and incubated for the next 30 min. DNS reagent (100 µL) was added and boiled at 80 °C for 20 min. The absorbance was measured at 450 nm in the plate reader. The blank was prepared with PBS buffer (100 mM, pH 6.8, 25 °C). The results are expressed as a percentage of enzyme inhibition.

The α-glucosidase inhibition assay was performed according to Tiwari et al. [24], with slight modifications. Flavonoid solutions (100 µL, 100 μM) were incubated with 50 µL of α-glucosidase (1 U/mL) for 10 min at 37 °C. Then, 50 µL of 5 mM 4-nitrophenyl α-D-glucopyranoside was added. After 5 min, absorbance was measured at 405 nm in the plate reader. The blank was prepared with the PBS buffer (100 mM, pH 6.8, 25 °C). The results are expressed as a percentage of enzyme inhibition.

### 2.5. Statistical Analysis

All analyses were performed in at least triplicate, and the results are expressed as mean ± standard deviation (SD). All statistical analyses were performed using PAST software (version 4.13) [25]. One-way ANOVA and post hoc multiple mean comparisons (Tukey’s HSD test) were performed, and differences between measurements were considered significant at *p* < 0.05.

## 3. Results

### 3.1. Chemical Formula of Investigated Standards

**Figure 3:**
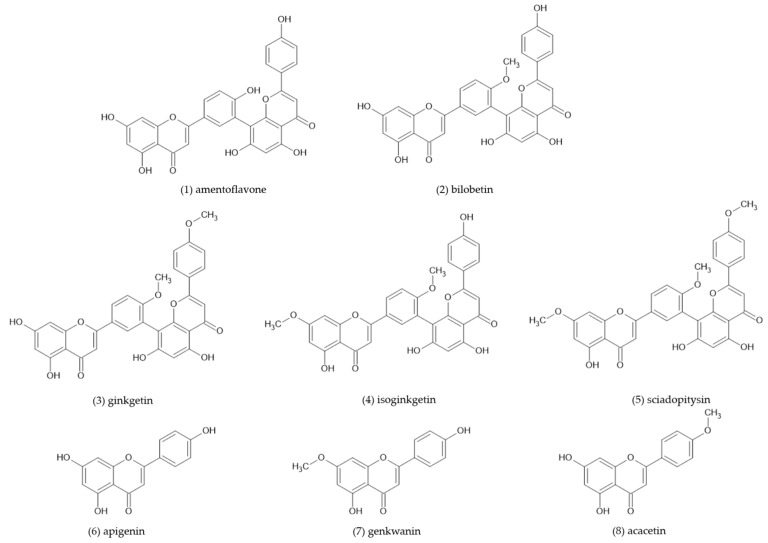
Chemical structure of investigated compounds.

Here, we studied five 3′-8″-biflavones (amentoflavone, bilobetin, ginkgetin, isoginkgetin, and sciadopitysin) and their monomeric subunits (apigenin, genkwanin, and acacetin), and their structures are shown in Figure 3. The number and position of methoxy and hydroxyl groups in their structures are summarized in Table 1.

As shown in Figure 3 and Table 1, all the dimers studied are dimers of the 3′-8″ type. Amentoflavone is a dimer of apigenin. The monomeric units of bilobetin are acacetin and apigenin, while isoginkgetin is an acacetin dimer. Ginkgetin consists of apigenin 4′,7-dimethyl ether and apigenin, whereas the monomeric subunits of sciadopitysin consist of apigenin 4′,7-dimethyl ether and acacetin. Unfortunately, apigenin 4′,7-dimethyl ether was not commercially available for inclusion in this study at the time the experiments were performed.

### 3.2. Antioxidant Activity

We measured antioxidant activity using the commonly used DPPH method, and the results are presented in Table 2.

Overall, all tested compounds showed very weak or no radical scavenging capacity, with no significant differences between them. The flavonoid quercetin, for example, showed 100% inhibition at the same concentration.

### 3.3. Antifungal Activity

In our experiment, we compared the antifungal activity against five fungi (Figure 2) at different concentrations and recorded the results at two wavelengths (Table 3).

As can be seen from the table, the antifungal activity depends on both the fungi used and the tested concentration. At the lowest concentration, 0.01 µg/mL, all the tested compounds showed significant inhibition only against *F. graminearum*, and the biflavonoids showed higher inhibition than the tested monomers. In the case of *F. verticillioides*, all biflavonoids and apigenin showed inhibition only at a concentration of 10 µg/mL, whereas at lower concentrations, they showed no inhibition or even slightly growth-promoting effects. In contrast, the antifungal activity against *A. ochraceus* was higher for all biflavonoids and genkwanin at lower concentrations and decreased with the increase in concentration. All biflavonoids showed antifungal activity against A. flavus, and the activity increased with concentration for most biflavonoids. Apigenin showed antifungal activity against A. flavus only at a concentration of 1 µg/mL, while genkwanin showed dose-dependent stimulatory effects on this fungus. In the case of *A. alternata*, antifungal activity was strongly concentration-dependent, but in general, biflavonoids showed weak activities, while apigenin and genkwanin showed no antifungal activity.

### 3.4. Enzyme Inhibition Activity

**Figure 4:**
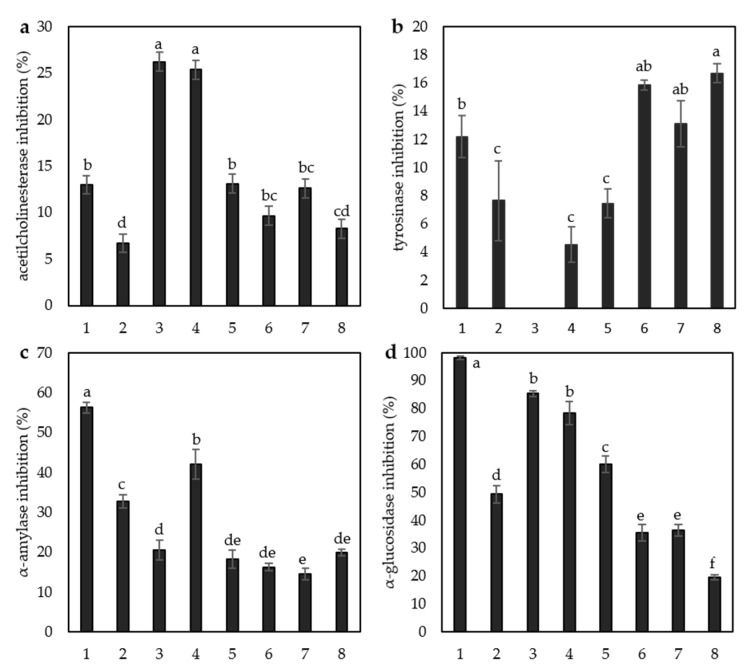
Enzyme inhibition activity of (1) amentoflavone, (2) bilobetin, (3) ginkgetin, (4) isoginkgetin, (5) sciadopitysin, (6) apigenin, (7) genkwanin, and (8) acacetin against (**a**) acetylcholinesterase, (**b**) tyrosinase, (**c**) α-amylase, and (**d**) α-glucosidase at a concentration of 100 μM. Values with different letters differ significantly at *p* < 0.05.

Enzyme inhibition activities of the compounds at 100 μM are shown in Figure 4. We measured inhibitory activities against four enzymes—acetylcholinesterase, tyrosinase, α-amylase, and α-glucosidase. Ginkgetin (26.24 ± 1.71%) and isoginkgetin (25.37 ± 0.66%) inhibited acetylcholinesterase significantly more than the other compounds tested. Tyrosinase inhibition activity was higher for the monomeric flavonoids acacetin (16.68 ± 0.67%), apigenin (15.85 ± 0.34%), and genkwanin (13.12 ± 1.65%) than for the dimeric flavonoids. Ginkgetin showed no inhibition against tyrosinase. The inhibition against α-amylase was highest with amentoflavone (56.26 ± 1.32%), followed by isoginkgetin (42.00 ± 3.68%) and bilobetin (32.76 ± 1.72%). Amentoflavone showed complete inhibition against α-glucosidase (98.17 ± 0.56%), and the other biflavonoids tested, ginkgetin (85.36 ± 1.06%), isoginkgetin (78.42 ± 4.15%), sciadopitysin (60.16 ± 2.90%), and bilobetin (49.36 ± 3.10%), also showed high inhibition compared with the monomeric flavonoids.

## 4. Discussion

Flavonoids are often considered antioxidants, but in reality, their antioxidant activity is highly dependent on the structure of individual flavonoids [26]. This is also evident from our results showing that flavones and biflavones have weak radical scavenging activity using one of the most commonly used methods for measuring antioxidant activity, DPPH. Similar to the results of our experiments, Kang et al. [27] revealed that none of the five biflavones also tested in our study showed radical scavenging activity up to 100 μM when measured with 1,1-diphenyl-2-picrylhydrazyl (DPPH). We tested even higher concentrations and observed no significant activity. This suggests that biflavones do not act as antioxidants or radical scavengers, at least in a cell-free system. Research findings also show that the biflavones amentoflavone, bilobetin, ginkgetin, and sciadopitysin are the weakest antioxidants among the 30 compounds isolated from ginkgo tested in myelomonocytic HL-60 cells [28]. However, there are some conflicting data on the antioxidant activity of biflavones. For example, Li et al. [29] compared the antioxidant activity of acacetin and its 3′,8″ dimer isoginkgetin using three methods, the O^2−^ scavenging assay, the Cu^2+^ reducing assay, and the 2,2′-azino-bis(3-ethylbenzothiazoline-6-sulfonic acid) radical scavenging assay, and reported that the 3′,8″-dimerization increased the antioxidant capacity of flavonoids. From all these findings, we can conclude that the antioxidant activity of biflavones is still very poorly understood, and further studies should clarify their activity as antioxidants.

Monomeric flavonoids have been shown to be effective antifungal agents against a variety of pathogenic organisms through mechanisms that include the disruption of the plasma membrane; the induction of mitochondrial dysfunction; and the inhibition of cell wall formation, cell division, RNA and protein synthesis, and efflux-mediated pumping [30]. Herein, we tested compounds for their potential inhibition against five mycotoxigenic fungi that are perhaps the most important pathogens of global concern in the context of food safety. They can affect the quality and quantity of marketable products by damaging foods such as corn, wheat, and peanuts and producing mycotoxin metabolites that can be carcinogenic and affect human and animal health [31]. According to our results, antifungal activity depends on both the fungi used and the tested concentration. The genus *Fusarium* generates a number of mycotoxins that can cause acute or chronic disease and, in some cases, death [32]. At the lowest concentration, 0.01 µg/mL, all the tested compounds showed significant inhibition against *F. graminearum*, and biflavonoids showed higher inhibition than the monomers analyzed. When we directly compared amentoflavone with its monomeric subunits apigenin, we found that amentoflavone had a stronger inhibition against *F. graminearum*, which may indicate that the 3′-8″-dimerization increases the antifungal activity against this fungus. In the case of *F. verticillioides*, all biflavonoids and apigenin showed inhibition at a higher concentration (10 µg/mL). Krauze-Baranowska and Wiwart [16] investigated the antifungal activity of amentoflavone, bilobetin, ginkgetin, and sciadopitysin against *F. culmorum*, and at a concentration of 100 μM/mL, bilobetin, ginkgetin, and sciadopitysin showed 100% inhibition. It has also been reported that the extracts of *Hypericum triquetrifolium* have antifungal activity against *Fusarium* sp. [33], and *Hypericum* sp. are known to contain biflavonoids [34]. Taken together, these findings indicate that the biflavonoids tested here can be potential inhibitors of *Fusarium* sp. Other mycotoxigenic fungi we tested belong to *Aspergillus* sp., namely *A. flavus* and *A. ochraceus*. All the tested biflavonoids, as well as genkwanin, showed antifungal activity against *A. ochraceus*, which was higher at lower concentrations and decreased with the increase in concentration, whereas for *A. flavus*, activity increased with concentration for most biflavonoids. Previously, isoginkgetin was reported to have inhibitory activity against *A. fumigatus* [35]. Gonçalez et al. [36] reported in their study that amentoflavone inhibited the production of aflatoxin B1 and B2 by *A. flavus* but did not inhibit fungal growth at the concentration tested. They indicated that biflavonoids may be effective agents for controlling aflatoxin production without inhibiting growth. All the compounds we tested here showed weak or no inhibition against *A. alternata* at concentrations up to 10 μg/mL. Krauze-Baranowska and Wiwart [16] investigated antifungal activity at a higher concentration of 100 μM and showed that ginkgetin had 100% inhibition against *A. alternata*, while the inhibition of amentoflavone, bilobetin, and sciadopitysin was 54%, 80%, and 59%, respectively.

For the enzyme inhibitory activities of our compounds, we selected four enzymes that are involved in important metabolic functions. Acetylcholinesterase (AChE) inhibitors are widely used for the symptomatic treatment of Alzheimer’s disease and other dementias because the inhibition of AChE slows the hydrolysis of acetylcholine and increases choline levels, which improves cognitive function. Monomeric flavonoids have been recognized as AchE inhibitors for several decades [37]. According to our results, at a concentration of 100 μM, ginkgetin and isoginkgetin had significantly higher inhibitory effects on acetylcholinesterase than the other compounds analyzed. These results are not surprising because ginkgetin and isoginkgetin are characteristic compounds of *Ginkgo biloba* L. [9,28,38], a plant used to treat cognitive disorders, and ginkgetin is known to be a potential neuprotectant [17]. The inhibition of tyrosinase is related to the potential use of compounds to reduce melanogenesis activity and alleviate hyperpigmentation [39]. According to our results, all three monomeric flavonoids, i.e., apigenin, genkwanin, and acacetin, showed significantly higher inhibitory activity than the biflavones studied. Among the biflavonoids, amentoflavone showed higher inhibitory activity. According to the molecular docking study by Ogunwa [40], amentoflavone has moderate tyrosinase inhibitory potential, which is also evident in our study.

We also investigated the inhibitory activity against α-amylase and α-glucosidase, the two enzymes involved in carbohydrate metabolism. The inhibition of these enzymes may help regulate blood glucose levels and prevent/control diabetes mellitus [23]. According to our results, amentoflavone is the best α-amylase inhibitor, followed by isoginkgetin. Ametoflavone has already been recognized as a potent α-amylase inhibitor, and its possible mechanism of action involves the occupation of the catalytic site and other regions of the enzyme as well as the inhibition of the substrate’s access and binding [40]. Peterson et al. [41] studied bilobetin, isoginkgetin, ginkgetin, and sciadopitysin isolated from a ginkgo for their α-amylase inhibitory activity. They found that bilobetin had no clear inhibitory effect, whereas, in the other components, the inhibitory effect decreased with the increase in concentration, and it was most pronounced for sciadopitysin, which they characterized as an α-amylase activator rather than an inhibitor. These results are consistent with our results where sciadopitysin showed the least inhibitory effect on α-amylase. In the case of α-glucosidase, amentoflavone showed complete inhibition under the concentration tested, followed by isoginkgetin and ginkgetin. Amentoflavone is already known to be a potent α-glucosidase inhibitor. Swargiary et al. [42] investigated α-glucosidase inhibitory activity via the molecular docking of 155 different phenolic compounds and found that amentoflavone had the strongest binding affinity with α-glucosidase, much stronger than the reference acarbose. Similarly, Li et al. [43] investigated the inhibitory effects of amentoflavone and monomeric apigenin on α-glucosidase and reported a stronger inhibitory effect of amentoflavone, which is also reflected in our results. Flavonoids are noncompetitive inhibitors of α-glucosidase and have shown synergistic inhibitory effects with acarbose [43], an α-glucosidase inhibitor used in conjunction with diet and exercise to control blood glucose levels in patients with type 2 diabetes mellitus.

## 5. Conclusions

In the present study, we evaluated the radical scavenging activity (with DPPH), antifungal activity against mycotoxigenic fungi (*A. alternata*, *A. flavus*, *A. ochraceus*, *F. graminearum*, and *F. verticillioides*), and enzyme inhibition (acetylcholinesterase, tyrosinase, α-amylase, and α-glucosidase) of five 3′-8″-biflavones (amentoflavone, bilobetin, ginkgetin, isoginkgetin, and sciadopitysin) and their monomeric subunits (apigenin, genkwanin, and acacetin). All the tested compounds showed weak radical scavenging activity. The antifungal activity strongly depends on the fungi used and the concentration. At some concentrations, we even detected growth-promoting activity, as in the case of *F. verticillioides*. At the lowest concentration, 0.01 µg/mL, all the analyzed compounds showed significant inhibition against *F. graminearum*, and the biflavonoids showed higher inhibition than the tested monomers. The antifungal activity against *A. ochraceus* was higher for all biflavonoids and genkwanin at lower concentrations and decreased with the increase in concentration, while all biflavonoids showed antifungal activity against *A. flavus*, and the activity increased with concentration for most biflavonoids. Biflavonoids showed weak activities against *A. alterata*, while apigenin and genkwanin showed no antifungal activity. Isoginkgetin and ginkgetin showed the highest inhibition against acetylcholinesterase, while monomeric compounds showed higher inhibitory activity against tyrosinase. Amentoflavone proved to be a potent inhibitor of α-amylase and α-glucosidase, and the 3′-8″-biflavones tested showed higher inhibitory activity against α-glucosidase than their monomeric subunits. From all these results, we can conclude that the 3′-8″-dimerization affects the biological activity, such as the antifungal activity against *F. graminearum*, where the biflavonoids showed higher inhibitory activity than the tested monomers. Also, the inhibitory activity against α-amylase and α-glucosidase is enhanced by 3′-8″-dimerization but is also influenced by the number of methoxy and hydroxyl groups.

## Figures and Tables

**Table 1:** Summarized information about the number and position of hydroxy and methoxy groups of investigated compounds.

	Hydroxy		Methoxy		Dimer
	Number	Position	Number	Position	Yes
Amentoflavone	6	4′, 4‴, 5, 5″, 7, 7″	0	0	Yes
Bilobetin	5	4′, 5, 5″, 7, 7″	1	4‴	Yes
Ginkgetin	4	4‴, 5, 5″, 7″	2	4′, 7	Yes
Isoginkgetin	4	5, 5″, 7,7 ″	2	4′, 4‴	Yes
Sciadopitysin	3	5, 7	3	4′, 4‴, 7″	Yes
Apigenin	3	4′, 5, 7	0	0	No
Genkwanin	2	5, 4′	1	7	No
Acacetin	2	5, 7	1	4′	No

**Table 2:** Radical scavenging activity of compounds at concentration 1 mg/mL.

	DPPH Radical Scavenging (% Inhibition)
Amentoflavone	4.60 ± 2.00 ^a^
Bilobetin	4.56 ± 0.97 ^a^
Ginkgetin	2.62 ± 1.41 ^a^
Isoginkgetin	2.99 ± 1.55 ^a^
Sciadopitysin	2.02 ± 00 ^a^
Apigenin	3.18 ± 1.28 ^a^
Acacetin	1.93 ± 1.27 ^a^
Genkwanin	3.01 ± 0.61 ^a^

Parameters sharing the same letter do not differ significantly at *p* > 0.05.

**Table 3:** Growth percentages of tested fungi grown under different flavonoid concentrations.

		*A. alternata*			
	Abs	0.01 μg/mL	0.1 μg/mL	1 μg/mL	10 μg/mL
**Amentoflavone**	**405**	96.39 ± 26.85	92.33 ± 37.88	83.00 ± 23.98	104.85 ± 10.71
	**450**	102.05 ± 27.28	93.65 ± 44.91	82.19 ± 28.09	70.11 ± 10.10
**Bilobetin**	**405**	79.53 ± 6.55	103.26 ± 33.66	67.67 ± 17.69	115.77 ± 21.30
	**450**	80.21 ± 6.82	106.76 ± 36.96	67.66 ± 21.07	91.20 ± 23.80
**Ginkgetin**	**405**	107.49 ± 31.25	88.63 ± 9.45	69.78 ± 6.57	98.68 ± 7.38
	**450**	113.31 ± 39.85	87.92 ± 9.65	68.07 ± 10.56	64.18 ± 13.33
**Isoginkgetin**	**405**	102.03 ± 10.01	103.96 ± 24.38	91.10 ± 13.17	96.92 ± 20.86
	**450**	106.96 ± 13.15	104.30 ± 25.93	93.04 ± 16.55	89.97 ± 40.77
**Sciadopitysin**	**405**	97.27 ± 19.89	92.51 ± 19.49	86.52 ± 21.03	85.64 ± 27.76
	**450**	101.64 ± 20.87	93.45 ± 22.60	87.92 ± 23.46	74.21 ± 30.19
**Apigenin**	**405**	106.96 ± 13.80	107.82 ± 30.62	105.76 ± 19.05	85.14 ± 21.09
	**450**	106.96 ± 13.80	107.82 ± 30.62	105.76 ± 19.05	85.14 ± 21.09
**Genkwanin**	**405**	133.42 ± 15.95	98.88 ± 29.52	103.35 ± 20.77	100.95 ± 24.86
	**450**	133.42 ± 15.95	98.88 ± 29.52	103.35 ± 20.77	100.95 ± 24.86
		***A. flavus***			
**Amentoflavone**	**405**	95.77 ± 14.78	80.90 ± 21.43	101.51 ± 20.18	87.77 ± 5.73
	**450**	98.44 ± 14.30	79.87 ± 21.30	100.00 ± 20.84	66.29 ± 5.07
**Bilobetin**	**405**	83.73 ± 23.45	77.05 ± 18.09	80.81 ± 15.21	85.70 ± 11.09
	**450**	84.37 ± 24.04	75.96 ± 18.67	78.11 ± 12.15	70.10 ± 12.13
**Ginkgetin**	**405**	123.49 ± 16.01	85.70 ± 26.01	67.36 ± 18.63	86.64 ± 13.77
	**450**	126.97 ± 13.37	87.20 ± 26.62	64.53 ± 18.92	68.83 ± 16.35
**Isoginkgetin**	**405**	87.86 ± 14.00	100.47 ± 14.07	85.79 ± 23.79	83.16 ± 10.27
	**450**	86.61 ± 16.26	100.88 ± 12.10	86.22 ± 24.90	70.69 ± 12.55
**Sciadopitysin**	**405**	83.73 ± 23.45	77.05 ± 18.09	80.81 ± 15.21	85.70 ± 11.09
	**450**	101.37 ± 15.36	70.59 ± 14.14	76.06 ± 18.01	64.92 ± 6.07
**Apigenin**	**405**	122.77 ± 0.68	98.32 ± 28.87	59.98 ± 12.89	116.06 ± 28.23
	**450**	127.93 ± 6.75	101.69 ± 32.22	57.88 ± 14.82	109.10 ± 26.78
**Genkwanin**	**405**	92.81 ± 12.75	108.39 ± 27.25	115.42 ± 26.45	134.51 ± 28.13
	**450**	92.15 ± 14.70	109.98 ± 28.23	115.97 ± 27.17	132.96 ± 36.58
		***A. ochraceus***			
**Amentoflavone**	**405**	90.39 ± 24.20	71.37 ± 14.29	70.17 ± 11.90	111.01 ± 9.84
	**450**	92.27 ± 23.16	73.99 ± 12.64	69.64 ± 13.19	74.65 ± 2.88
**Bilobetin**	**405**	82.18 ± 4.95	67.57 ± 18.80	85.19 ± 12.11	131.63 ± 19.62
	**450**	80.96 ± 4.05	68.77 ± 19.37	87.49 ± 12.65	111.86 ± 24.49
**Ginkgetin**	**405**	96.20 ± 43.63	80.78 ± 22.13	80.38 ± 25.30	117.02 ± 9.44
	**450**	102.50 ± 45.50	82.92 ± 19.64	81.39 ± 23.17	79.43 ± 14.64
**Isoginkgetin**	**405**	75.38 ± 10.93	82.78 ± 18.20	83.98 ± 9.58	101.40 ± 14.93
	**450**	80.74 ± 10.97	84.66 ± 18.28	86.40 ± 8.45	90.32 ± 18.62
**Sciadopitysin**	**405**	84.58 ± 19.27	75.98 ± 10.35	75.98 ± 12.08	101.40 ± 13.16
	**450**	84.87 ± 19.79	74.43 ± 9.77	78.13 ± 14.20	86.62 ± 15.69
**Apigenin**	**405**	75.98 ± 30.47	67.60 ± 18.56	66.20 ± 14.16	54.26 ± 8.16
	**450**	78.24 ± 31.73	65.65 ± 16.83	67.31 ± 14.24	61.39 ± 24.44
**Genkwanin**	**405**	77.38 ± 26.74	59.56 ± 26.28	69.34 ± 15.73	116.51 ± 21.53
	**450**	78.06 ± 27.16	58.80 ± 25.89	67.31 ± 14.62	83.06 ± 9.30
		***F. graminearum***			
**Amentoflavone**	**405**	67.99 ± 20.62	68.89 ± 19.25	47.01 ± 7.74	83.00 ± 15.23
	**450**	58.20 ± 22.27	63.86 ± 29.56	38.72 ± 10.99	61.55 ± 18.03
**Bilobetin**	**405**	58.22 ± 6.52	76.70 ± 15.94	64.49 ± 20.76	90.91 ± 27.33
	**450**	55.16 ± 10.09	71.18 ± 18.64	62.19 ± 23.21	76.45 ± 28.66
**Ginkgetin**	**405**	54.99 ± 18.02	63.59 ± 12.16	57.59 ± 20.08	89.51 ± 21.64
	**450**	44.71 ± 23.54	53.71 ± 16.45	55.51 ± 25.52	67.97 ± 28.65
**Isoginkgetin**	**405**	48.48 ± 7.01	69.39 ± 10.40	61.29 ± 10.61	65.89 ± 14.61
	**450**	35.98 ± 12.50	55.25 ± 18.99	58.85 ± 12.44	57.43 ± 19.33
**Sciadopitysin**	**405**	52.62 ± 8.64	72.30 ± 20.12	55.82 ± 4.01	80.20 ± 25.92
	**450**	53.28 ± 15.54	68.23 ± 22.74	53.79 ± 8.48	69.00 ± 34.25
**Apigenin**	**405**	81.36 ± 22.43	79.16 ± 27.67	83.51 ± 26.00	92.09 ± 10.01
	**450**	78.58 ± 37.50	83.23 ± 43.84	74.05 ± 26.50	62.75 ± 12.56
**Genkwanin**	**405**	77.92 ± 22.72	74.43 ± 18.08	84.51 ± 19.32	114.73 ± 31.86
	**450**	70.70 ± 21.95	67.87 ± 26.52	83.05 ± 28.45	105.83 ± 29.53
		***F. verticillioides***			
**Amentoflavone**	**405**	104.84 ± 5.45	95.39 ± 19.55	102.31 ± 5.71	82.67 ± 6.20
	**450**	109.03 ± 7.77	100.16 ± 23.97	104.59 ± 5.74	78.16 ± 8.28
**Bilobetin**	**405**	103.38 ± 12.66	101.63 ± 9.10	111.53 ± 13.48	70.16 ± 4.84
	**450**	105.90 ± 13.52	105.53 ± 9.75	112.96 ± 15.25	66.31 ± 6.04
**Ginkgetin**	**405**	103.26 ± 11.04	113.67 ± 19.59	123.12 ± 15.76	80.08 ± 6.72
	**450**	109.28 ± 12.94	120.90 ± 23.15	129.52 ± 17.86	76.10 ± 7.43
**Isoginkgetin**	**405**	110.97 ± 12.60	101.91 ± 7.77	117.78 ± 6.58	84.36 ± 8.42
	**450**	115.71 ± 16.08	106.53 ± 11.35	122.21 ± 7.83	83.04 ± 7.26
**Sciadopitysin**	**405**	107.88 ± 20.54	97.02 ± 5.27	102.53 ± 18.36	71.70 ± 8.89
	**450**	114.34 ± 24.68	100.53 ± 6.77	107.40 ± 20.60	71.35 ± 10.72
**Apigenin**	**405**	122.94 ± 11.65	102.53 ± 8.07	121.55 ± 16.24	88.68 ± 9.07
	**450**	132.06 ± 11.84	106.53 ± 9.13	128.44 ± 19.07	93.27 ± 10.02
**Genkwanin**	**405**	98.07 ± 25.09	107.89 ± 19.90	105.42 ± 5.58	105.48 ± 3.18
	**450**	114.35 ± 24.59	113.63 ± 21.19	108.34 ± 6.71	103.72 ± 7.75

## Data Availability

Additional data are available upon request.
